# Initial Results of Tests Using GSR Biofeedback as a New Neurorehabilitation Technology Complementing Pharmacological Treatment of Patients with Schizophrenia

**DOI:** 10.1155/2021/5552937

**Published:** 2021-06-10

**Authors:** Renata Markiewicz, Beata Dobrowolska

**Affiliations:** ^1^Psychiatric Nursing Department, Faculty of Health Sciences, Medical University of Lublin, Poland; ^2^Department of Management in Nursing, Faculty of Health Sciences, Medical University of Lublin, Poland

## Abstract

Galvanic skin response (GSR) Biofeedback uses training to reduce tension and anxiety and improve concentration and self-regulation. The study was aimed to evaluate this method as a form of rehabilitation and quantify the outcomes achieved by patients undergoing training using this technique. Six schizophrenic patients were enrolled in the study and underwent training based on the relaxation training module (CENTER), concentration training module (BALANCE), and self-regulation training module (INSECTS). Training sessions were held twice a week for 6 weeks. From the total group of subjects involved in the study, two patients had a statistically significant increase in measured values after the CENTER exercise, indicating that relaxation was achieved. Four patients showed a statistically significant decrease in measured values after the BALANCE exercise, which was reflective of an improvement in concentration. Three patients had a statistically significant decrease in measured values after the INSECTS exercise, which indicated an improvement in self-regulation. GSR Biofeedback may be used to complement the pharmacological treatment of patients diagnosed with schizophrenia.

## 1. Introduction

Schizophrenia is one of the most common causes of disability in young people [1]. Clinical diagnosis of schizophrenia requires finding specific clinical symptoms, persisting for a specific time. Eight dimensions of schizophrenia have been distinguished in the Diagnostic and Statistical Manual of Mental Disorders Fifth Edition (DSM-5): hallucinations, delusions, speech disorganisation, abnormal psychomotor behaviour, negative symptoms, cognitive disorders, depression, and mania [2].

Cognitive deficits dominating in schizophrenia patients are associated mainly with disorganisation and negative symptoms. Because they are chronic, they negatively affect the patients' overall social life [3–5]. Research shows that cognitive deficits are now present regardless of the disease stage and mainly concern functional disorders in the frontal and temporal lobes [6–13].

Despite multidirectional research, it is still difficult to clearly determine what factors are decisive for maintaining health or preventing the occurrence of disease in schizophrenia patients. It appears that the sense of coherence (SOC), a concept proposed by Antonovsky (a salutogenic model which assumes that health can only be preserved when there is a balance between stress and coping. This process is affected by biological factors, stressors, lifestyle, and coherence, all of which comprise an individual's interactions with the environment) is a crucial component in the recovery process and components such as a sense of comprehensibility, manageability, and meaningfulness fully explain this concept [14].

From the point of view of schizophrenia patients, Antonovsky's concept proves to be interesting because it concerns general life orientation and covers all areas associated with correct reception of external and internal information, activity, and personal resources. This salutogenic model plays an important role in coping with stress and problems in daily life.

Increasingly, new possibilities are being sought that, based on therapeutic interventions, would enable the reduction of deficits resulting from the disease. Today, much interest is focused on neuronal mechanisms, which, being physiological markers, can be used to guide treatment decisions regarding existing deficits. One such indicator is electrodermal activity (EDA), which provides information about emotions, cognitive processes, and behaviour, and hence also about the functioning of different brain areas. The galvanic skin response (GSR), both the general tonic-level electrodermal component (skin conductance level, SCL) and the phasic component (skin conductance responses, SCR_s_), is a measure used in the diagnosis and treatment of mental disorders, and Biofeedback training method based on GSR measurements allows to modulate a patient's emotional state as the need arises [15].

The ability to reduce stress presumably increases the patients' sense of coherence, protects them against relapse, enhances their cognitive processes, and improves their quality of life. Such a possibility is offered by GSR Biofeedback (GSR BF), which uses specially selected exercises to reduce the level of tension and anxiety and improve concentration and self-regulation. Importantly, the program provides an interesting interactive interface between the patient and the computer. The effects of this method are confirmed by numerous studies which describe a wide range of applications of this method in the rehabilitation of mentally ill patients with anxiety disorders [16–18], depression [19, 20], suicidal tendencies [21, 22], bipolar affective disorder [23, 24], and schizophrenia [25–27]. By analysing the electrodermal activity signal on baseline, phasic SCR_s_, and tonic SCL data, one can determine the phase of the disease, severity of symptoms, and the possibility of relapse. These extremely sensitive indicators reveal many features that are common to specific clinical entities, which, from the diagnostic point of view, are important for further treatment decisions [15].

Today, there are many studies available regarding the use of neurofeedback in the rehabilitation of people with mental disorders [28–31]. In addition, there is a lively discussion about the effectiveness of this method in psychiatry, and at the same time, it is recommended to continue research on this topic [32, 33]. While research on the meaning of the galvanic skin response in groups of patients with schizophrenia was carried out and the pioneer of these studies was Gruzelier [34, 35], currently, studies using GSR are not widely conducted. However, it is an important neurophysiological indicator as well as muscle (EMG) and brain (EEG) indicators.

This study was aimed to evaluate the GSR Biofeedback method as a form of rehabilitation and to demonstrate the outcomes achieved by schizophrenia patients undergoing training based on three modules: relaxation (CENTER), concentration (BALANCE), and self-regulation (INSECTS).

## 2. Materials

### 2.1. Characteristics of the Studied Group

The average patient age was 41 years (range: 34-45 years, median: 42.5); four patients were single and two were divorced. All patients came from large cities (over 100 thousand inhabitants); all were receiving a disability allowance and lived alone or with their parents. Three subjects had completed primary education (until 15 years of age), while the other three had completed secondary education (until 18 years of age). The mean number of subjects' hospitalisations was 8 stays (range: 4-15; median: 7) and was possibly associated with irregular treatment, alcohol consumption, or discontinuation of medication. The mean age at the time of the first hospitalisation was 21 years (range: 22-29; median: 24). Four patients did not report suicidal attempts and self-harm; two patients did report such episodes. All subjects were right-handed, took atypical neuroleptics, had no neuroleptic malignant syndrome (NMS) complications, and had no family history of schizophrenia; no drug allergies were reported; none had undergone major surgery; and none had problems with alcohol addiction. These details are presented in Table 1.

## 3. Method

### 3.1. Design

Six schizophrenia patients in remission were included in the pilot study. Considering the main deficits, which patients experience in schizophrenia [1, 3, 4, 8–14] standard training based on the relaxation (CENTER), concentration (BALANCE), and self-regulation (INSECTS) modules on the Elmiko Digi-Track apparatus (Elmiko-Medical Company, Warsaw, Poland) was organised. Convenience sampling was used to recruit participants, who were patients of psychiatric day ward in one hospital in the eastern part of Poland and had to meet the following study inclusion criteria: a patient's informed consent, a clinical diagnosis of schizophrenia (DSM-V), age (18–50 years), right-handedness, no history of neurological diseases, and exclusion of mental impairment, dementia, and addiction to alcohol. To ensure anonymity, patients were given codes consisting of two letters BF (short for Biofeedback) and a number: BF-2, BF-3, BF-7, BF-8, BF-9, BF-10.

### 3.2. Procedure

The participants who did the CENTER exercise were instructed to bring bubbles appearing on the screen into a circle in the centre of the screen; the faster the person relaxed, the faster they placed the bubbles in the circle. Participants practising in the BALANCE module had the task of placing and holding a ball in the middle of a tilting board; correct performance of the task testified to increased activation. The exercise in the INSECTS module consisted of turning ants running across the screen into ladybugs by clicking on them with the mouse; correct performance showed that a balance in self-regulation had been achieved.

Each training module was analysed for the level of the participants' relaxation and activation. The results were analysed using a PC and included the following indicators: average, baseline SCL value (start value), maximum relaxation value (expressed as a percent or as an absolute value in kOhm), and the minimum relaxation value, which was also the maximum activation value (expressed as a percent or as an absolute value in kOhm) [36].

The initial/start value was calculated automatically and depended on the participant's level of activation at the beginning of the session (dashed line). The computer registered the patient's psychophysical state during each new session relative to the start value. The average was represented by a solid line which connected data points representing average relative changes in GSR. The relative position and distance between the two lines depended on the participant's initial activation state and the maximum and minimum values of relaxation and activation. The dashed line positioned above the solid line meant that the participant showed higher activation (a higher start value), which should decrease during the session. A decrease in the value associated with activation (solid line below dashed line) indicated an increase in the level of relaxation, and the data points connected by the solid line were averages of the values obtained during the whole session. A declining solid line with a small number of phasic activities (SCR_s_) indicated that relaxation dominated over activation. Conversely, a rising solid line and an increased number of SCR_s_ showed that activation dominated over relaxation. The main goal of all the training sessions was to stabilize the participants' mental state and control and modulate it depending on their current psychophysical condition. Exercises in the three modules allowed to alleviate the complaints reported by the patients (they enhanced cognitive processes; improved concentration, memory, perceptiveness, and executive functions; and stabilized emotions).

**Figure 1:**
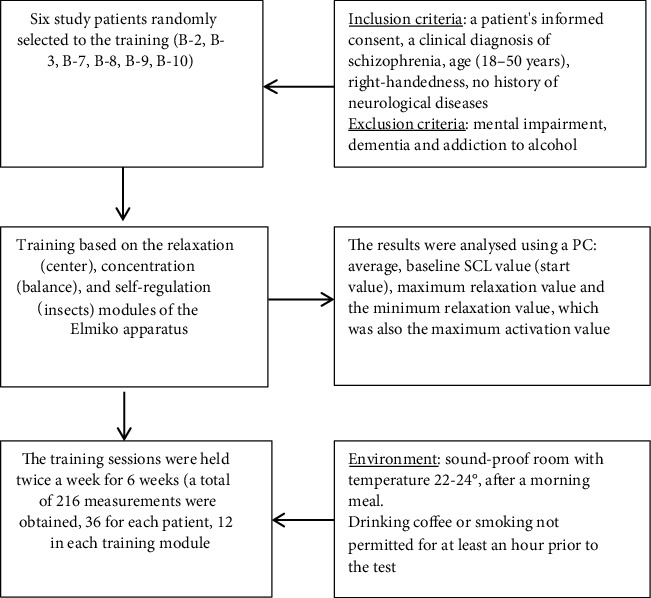
Research procedure.

The tests were performed in accordance with the adopted schedule. Measurements were made with electrodes attached to fingers (index and ring) of the left hand, coupled to a device which displayed the sequential training modules. The training time was determined by the computer, in accordance with the Elmiko Digi-Track apparatus (Elmiko-Medical Company, Warsaw, Poland) Instruction Manual, and the patients' results were recorded in graphic form at the end of each session. Demographic data were collected using a proprietary questionnaire, and hemispheric dominance was determined on the basis of Linksman's test [37] (Figure 1).

### 3.3. Ethical Issues

The subjects had given their consent to participate in the exercises and were thoroughly acquainted with the procedure and the training method. They also provided a written statement on participation in the experiment. The study protocol was accepted by the Bioethics Committee of the Medical University of Lublin—approval no. KE-0254/35/2019.

### 3.4. Analysis

Statistical analyses were performed using the Statistica 9.1 package (StatSoft Polska). The study procedure included an analysis of changes in the initial values (I.V.) and mean values (M.V) for measurements obtained during sessions conducted in each module; the significance of the changes was determined using the Wilcoxon signed-rank test.

(1) $$\begin{array}{c} z=\frac{T-n\left(n+1\right)/4}{\sqrt{n\left(n+1\right)\left(2n+1\right)/24}-\sum t^{3}-\sum^{t}/48}, \end{array}$$

∑ R_+_ is the sum of positive ranks. ∑ R_-_ is the sum of negative ranks.

The trend was established on the basis of the mean values of the initial measurements for each training day using the Spearman rank correlation coefficient.

(2) $$\begin{array}{c} r_{s}=1-\frac{6\sum_{i=1}^{n}d_{i}^{2}}{n\left(n^{2}-1\right)}, \end{array}$$

*d* ^2^_*i*_ is the squares of the differences between the ranks of the corresponding values of the *x*_*i*_ and *y*_*i*_ characteristics. *n* is the number of data pairs (number of rows in the table).

## 4. Results

The tests were performed using three similar modules in accordance with the adopted schedule, at the same time intervals for all patients. The values measured in the studied group and the significance of the change between I.V. and M.V. are presented in Table 2.

The analyses were based on 12 measurements for each patient. For patient BF-2, no statistically significant changes in the CENTER measurements (*Z* = 1.020, *p* = 0.308) were observed during the exercise, but such changes did occur when the patient did the BALANCE exercise (*Z* = 2.589, *p* = 0.010), in which a decrease was visible in the measured values, indicating activation. For the INSECTS module, statistically significant changes were noted (*Z* = 2.490, *p* = 0.013) during the exercise, associated with a reduction in measured values. The above data suggest that the patient did not achieve relaxation when training with the CENTER module but did achieve an improvement in concentration and self-regulation when doing the BALANCE and INSECTS exercises, respectively. Also, in case of patient BF-3, no statistically significant changes in the CENTER measurements were noted (*Z* = 1.336, *p* = 0.182) during the exercise, but such changes did occur when the BALANCE module was used (*Z* = 2.275, *p* = 0.023), for which a decrease was visible in the measured values, indicating activation. Statistically significant changes were also noted (*Z* = 2.118, *p* = 0.034) during the INSECTS exercise, which were associated with a reduction in measured values. The above data suggests that the patient did not achieve relaxation during the exercises using the CENTER module but did achieve an improvement in concentration and self-regulation when training in the BALANCE and INSECTS modules, respectively. Similarly, in case of patient BF-9, no statistically significant changes in the CENTER measurements were observed (*Z* = 0.078, *p* = 0.937) during the exercise, in contrast to the BALANCE exercise (*Z* = 2.275, *p* = 0.023), during which a decrease in the difference in values, representing activation, was noted. Statistically significant changes, associated with a reduction in the measured values, were also found for the INSECTS module (*Z* = 2.275, *p* = 0.023). The above data suggests that during the exercises in the CENTER module, the patient did not achieve relaxation, but training in the BALANCE and INSECTS modules improved the patient's concentration and self-regulation.

In the case of patient BF-7, statistically significant increases in the CENTER measurements (*Z* = 2.746, *p* = 0.006) and the BALANCE measurements (*Z* = 3.059, *p* = 0.002) were observed during training, which indicated that relaxation and an improvement in concentration were achieved. For the INSECTS module, no statistically significant changes were noted (*Z* = 1.922, *p* = 0.055) during the exercise, which meant that the patient achieved lower values of the parameters measured, which pointed to problems with self-regulation. For patient BF-8, statistically significant changes were noted in the CENTER measurements (*Z* = 2.667, *p* = 0.008) during exercises, but no such changes were seen for the BALANCE (*Z* = 0.157, *p* = 0.875) and INSECTS (*Z* = 1.334, *p* = 0.182) modules. The above data indicates that during the BALANCE and INSECTS exercises, the patient achieved lower values of the parameters measured, which were indicative of problems with self-regulation and concentration. The higher differences in measured values for the CENTER module are suggestive of increased relaxation.

In case of patient BF-10, no statistically significant changes during training were noted in the CENTER (*Z* = 0.549, *p* = 0.583), BALANCE (*Z* = 0.275, *p* = 0.784), and INSECTS (*Z* = 1.451, *p* = 0.147) measurements. The above data indicates that the parameters measured increased during training in the CENTER and BALANCE modules, indicating that the patient had problems with relaxation and concentration, respectively. A decrease in the measured values during the INSECTS exercise was reflective of problems with self-regulation.

The obtained data imply that in two out of the six patients, the values of the parameters measured increased significantly after the CENTER exercise, which means that the exercise helped the subjects relax. In four out of the six patients, the values of the parameters measured decreased significantly after exercises in the BALANCE module, which indicated an improvement in concentration (reduced relaxation). In three out of the six patients, the values of the parameters measured decreased significantly after the INSECTS exercise, showing that it had a positive effect on the level of self-regulation. Details regarding the effects of the therapy together with the type of changes observed are shown in Table 3.

The data shows that during the GSR Biofeedback training, measurements recorded in 71% of the patients were associated with relaxation in the CENTER module, in 54% of the patients the measurements were associated with activation in the BALANCE module, and in 54% of the patients, they were associated with activation in the INSECTS module. Activation and relaxation were understood as any, even the smallest, change in the measured values.

In a further part of the study, a trend determined on the basis of the mean initial measurement values (I.V.) calculated at the beginning of each session on a given day of experiment was analysed. Spearman's correlation coefficient was used to evaluate the trend measured (Table 4). The analysis indicates that there is a statistically significant increasing trend in four (out of the six) randomly studied patients (BF-2, BF-3, BF-7, BF-9). The positive correlation coefficient indicates that with the successive measurements, the mean initial measurement value (I.V.) increased.

**Figure 2:**
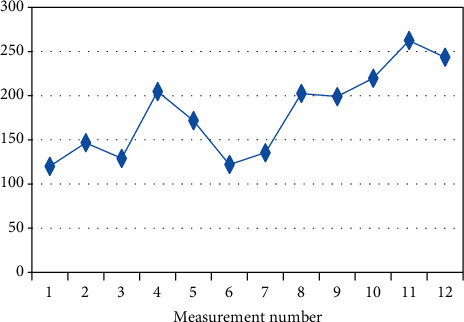
Changes in the mean measurement values (I.V.) in patient BF-2 (*R*_*s*_ = 0.769, *p* = 0.003) on a given day.

**Figure 3:**
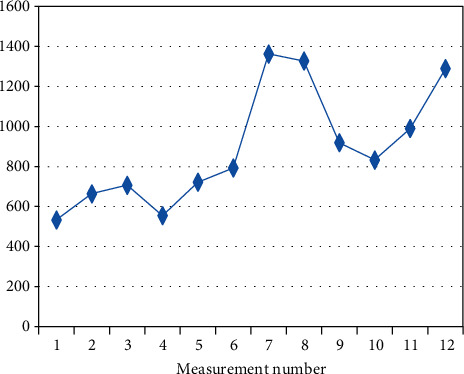
Changes in the mean measurement values (I.V.) in patient BF-3 (*R*_*s*_ = 0.797, *p* = 0.002) on a given day.

**Figure 4:**
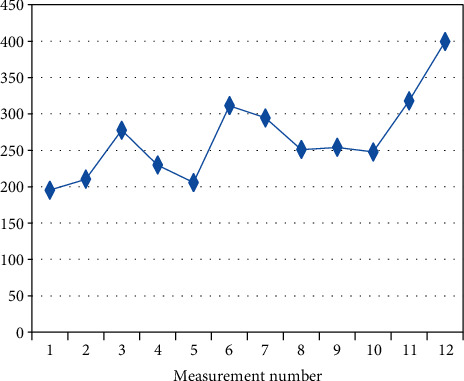
Changes in the mean measurement values (I.V.) in patient BF-7 (*R*_*s*_ = 0.692, *p* = 0.013) on a given day.

**Figure 5:**
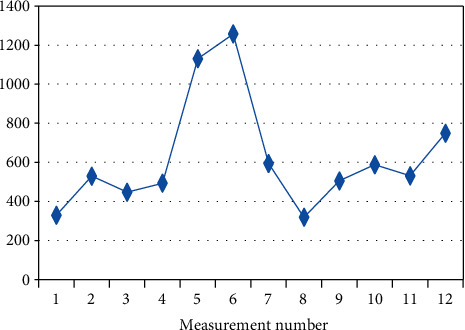
Changes in the mean measurement values (I.V.) in patient BF-8 (*R*_*s*_ = 0.364, *p* = 0.245) on a given day.

**Figure 6:**
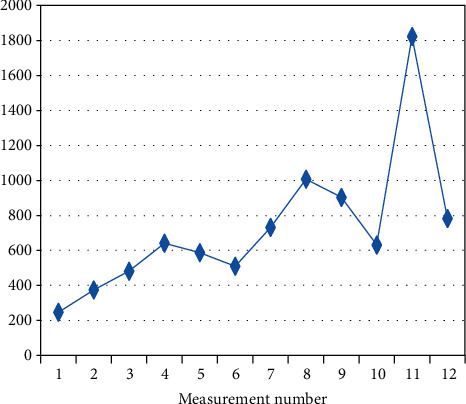
Changes in the mean measurement values (I.V.) in patient BF-9 (*R*_*s*_ = 0.825, *p* = 0.001) on a given day.

**Figure 7:**
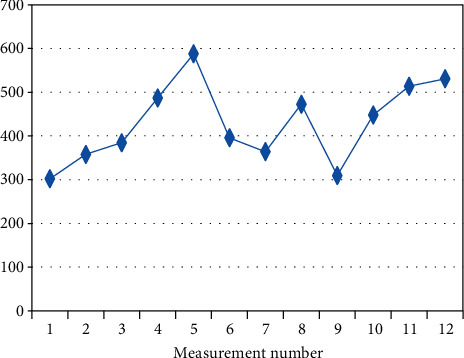
Changes in the mean measurement values (I.V.) in patient BF-10 (*R*_*s*_ = 0.483, *p* = 0.112) on a given day.

The figures below present changes in the mean initial values of I.V. measurements in the individual patients on a given day (Figures 2–7).

## 5. Discussion

The present preliminary study of GSR Biofeedback as a neurorehabilitation method shows that it is an interesting option that might be employed to supplement pharmacotherapy in schizophrenia patients. As the initial results indicate, the therapy effectively improves cognitive processes, which implies that it can also improve concentration and self-regulation, as well as reducing tension, anxiety, and fear. The obtained data based on the self-regulation process are confirmed by other authors, including Birbaumer et al. [38] and Mathiak et al. [39], who compared this process to a mechanism associated with learning and instrumental conditioning based on reinforcing and rewarding specific behaviours. Those authors are of the opinion that the dopaminergic system is involved in the coding of the reward pathway (substantia nigra—SN/tegmental area—VTA), playing an important role in self-regulation during the whole training process.

A similar opinion was presented by Sulzer, who emphasised a positive correlation between SN/VTA regulation, skin conductance (GSR), and emotional stimulation. According to this author, this correlation provides evidence for a positive effect of the therapy and a “control over secretion of endogenous dopamine” [40]. Stoeckel et al. (2014) are of the opinion that the appropriately selected Neurotherapy methods are a prerequisite for improvement in cognitive functions, “inducing a process of the brain function transformation” and influencing overall functioning [41]. Koush and colleagues state that “brain training” can be understood as positively acquired behavioural feedback improving psychical function and developing the functional network of the brain through visual exercises [42].

The high effectiveness of Biofeedback therapy is also emphasised by other authors, who note that regulation of brain waves based on regular training reduces fear, anxiety, and stress levels, that is, those symptoms which are frequently a problem for patients with mental disorders [43–45]. It is possible that this relationship is an effect of mental status modulation, and the process of instrumental conditioning is associated with morphological and functional changes in dendrites and neuroplasticity of the brain [46].

The preliminary results presented in this paper appear to confirm this process, as four (out of the six) participants subjected to training using GSR Biofeedback, obtained positive therapy outcomes, and the statistically significant increasing trend showed that the mean initial value (I.V.) of the measurements increased with each successive training session. The study indicates that in two out of the six subjects, a statistically significant increase in the measured values occurred during exercises in the CENTER module, which indicated that relaxation was achieved by those patients. In three patients, a statistically significant drop in measured values was noted after they had done the BALANCE exercise, which was reflective of an improvement in concentration. In three patients, a statistically significant drop in measured values was noted after the INSCECTS exercise, which was indicative of an improvement in self-regulation.

Possibly, each successive exercise performed in the individual modules resulted in regular improvement, and changes in action potentials (sprouting process) released the so-called priming process (long-term potentiation—LTP) and a sequential cycle for the formation of new chemical synaptic connections. In consequence, the cyclic training sessions led to positive therapy outcomes [44, 46, 47].

The positive effect of the therapy confirms the inverse relationship between the central and the autonomic activities, as discussed, among others, by Lim. The author noted a negative correlation between the amplitude of SCR_s_ and the amplitudes of the N200 potential and of *alpha* and *beta* waves [45]. A similar opinion is presented by Campo [27], who found a negative correlation between SCR_s_ and exacerbation of schizophrenia symptoms (positive and negative), and by Iacono [48], who observed a relationship between electrodermal activity and the *alpha* (drop) and *delta* (increase) rhythms. This inverse correlation indicates that modulation of the patient's mental condition based on GSR Biofeedback influences cortical transformation, and the positive effect of the therapy manifests as the reduction of adverse symptoms and improvement in coherence, as demonstrated in the present preliminary study.

Our study has some limitations which are mostly related to the model of apparatus which was used in GSR Biofeedback training and limited electrodermal measurements possible. Other limitations in this study include the small sample size and the use of convenience sampling methodology.

## 6. Conclusions

According to the results of this present pilot study, GSR Biofeedback may be considered as a new neurorehabilitation technique that could complement pharmacological treatment of patients diagnosed with schizophrenia, improving their individual capabilities and social functioning using their own resources.

Undoubtedly, further studies in a larger cohort are needed to formulate sound and consistent conclusions. The present study was a pilot experiment, but it already provided promising results, which warrant further investigation. From the point of view of the mentally ill patient, every method that can improve their social functioning and quality of life is worth exploring.

## Figures and Tables

**Table 1:** Demographic data and data associated with the patients' disease.

Variable		*n*
Place of residence	Large city (above 100 thousand)	6
	City (below 100 thousand)	0
	Village	0
Living situation	Alone	3
	With parents	3
	With family	0
Education	Primary	3
	Vocational	0
	Secondary	3
	University	0
Employment	In profession	0
	Outside profession	0
	Unemployed	0
	Benefit (awarded for a definite period)	1
	Disability benefit (awarded for an indefinite period)	5
Marital status	Single	4
	Married	0
	Divorced	2
	Widower	0
Number of children	None	4
	One child	2
Family history—mother	No	6
	Yes	0
Family history—father	No	6
	Yes	0
Outpatient monitoring	Regular (once a month)	4
	Irregular (less often than once a month)	2
	None	0
Self-mutilation	None	4
	Surface cuts	1
	Deep cuts	1
	Other	0
Suicide attempts	No	4
	Yes	2
Causes of disease recurrence	Alcohol (occasional consumption)	2
	Withdrawal of medicines	3
	No clear reason	1
Substance use	None	3
	Alcohol	0
	Drugs	0
	Nicotine	3
	Other	0
Medication	Atypical	6
	Typical	0
	Mixed	0
NMS	No	6
	Yes	0
Allergies to medicines	No	6
	Yes	0
Total		6

**Table 2:** Values measured in the studied group and significance of the change between I.V. and M.V.

Patient: CODE	Method	Measurement	Average	Median	Min	Max	Bottom quartile	Upper quartile	Standard deviation
BF-2	CENTER	I.V.	160.17	128.5	79	329	101	220	77.41
		M.V.	169.33	135	64	322	94.5	264	92.79
		Difference	9.17	6	-39	53	-10	28	27.29
		Difference significance	*Z* = 1.020, *p* = 0.308						
	BALANCE	I.V.	160.92	149	82	313	101.5	193.5	70.28
		M.V.	153.67	142	63	315	93.5	191.5	72.63
		Difference	-7.25	-7	-19	4	-12.5	-2	7.44
		Difference significance	*Z* = 2.589, *p* = 0.010						
	INSECTS	I.V.	234.58	226.5	103	370	202.5	286	78.60
		M.V.	166.00	163.5	87	275	110	216.5	62.73
		Difference	-51.92	-37	-163	39	-104	-2	63.60
		Difference significance	*Z* = 2.490, *p* = 0.013						
BF-3	CENTER	I.V.	772.00	734.5	504	1458	600.5	801	273.00
		M.V.	841.17	742	508	1457	622.5	982.5	308.65
		Difference	69.17	41	-78	251	-26	205	124.16
		Difference significance	*Z* = 1.336, *p* = 0.182						
	BALANCE	I.V.	941.67	843	508	1831	717	1034.5	394.96
		M.V.	859.17	759	496	1695	597	978.5	368.79
		Difference	-82.50	-94	-253	30	-143	-0.5	88.72
		Difference significance	*Z* = 2.275, *p* = 0.023						
	INSECTS	I.V.	1293.00	1122	552	2624	912.5	1620	559.87
		M.V.	1066.75	925.5	484	2300	777	1333	486.40
		Difference	107.50	175.5	-180	302	-6.5	212	144.07
		Difference significance	*Z* = 2.118, *p* = 0.034						
BF-7	CENTER	I.V.	310.58	265.5	159	636	217.5	398.5	139.31
		M.V.	400.67	393	207	665	277	515	154.62
		Difference	90.08	81.5	-45	227	35.5	145.5	75.17
		Difference significance	*Z* = 2.746, *p* = 0.006						
	BALANCE	I.V.	220.17	220	115	353	187	250.5	64.36
		M.V.	285.33	300.5	148	376	253	334	74.72
		Difference	65.17	49	11	149	21.5	103	49.06
		Difference significance	*Z* = 3.059, *p* = 0.002						
	INSECTS	I.V.	377.00	362.5	229	638	301.5	449	111.28
		M.V.	309.50	296.5	201	519	230	376	92.37
		Difference	41.58	49.5	-73	175	7	77.5	67.34
		Difference significance	*Z* = 1.922, *p* = 0.055						
BF-8	CENTER	I.V.	467.58	387	221	1381	306.5	481	307.96
		M.V.	511.58	425.5	247	1423	334	598	315.18
		Difference	44.00	37.5	-17	161	13.5	54.5	50.59
		Difference significance	*Z* = 2.667, *p* = 0.008						
	BALANCE	I.V.	612.58	512	194	1654	397	711.5	374.80
		M.V.	611.83	535.5	227	1662	385	710.5	371.79
		Difference	-0.75	20.5	-320	260	-75	58.5	142.49
		Difference significance	*Z* = 0.157, *p* = 0.875						
	INSECTS	I.V.	815.83	663.5	295	2698	496.5	869	625.78
		M.V.	726.42	684	184	2267	452	755.5	524.72
		Difference	-62.83	-78	-416	171	-119.5	74.5	156.95
		Difference significance	*Z* = 1.334, *p* = 0.182						
BF-9	CENTER	I.V.	663.58	484.5	120	1967	350.5	799.5	530.65
		M.V.	613.58	536.5	130	1828	388.5	690	440.82
		Difference	-50.00	20	-649	171	-109.5	54.5	211.32
		Difference significance	*Z* = 0.078, *p* = 0.937						
	BALANCE	I.V.	767.42	510.5	250	1849	434	990.5	554.76
		M.V.	698.58	501	243	1759	423.5	776.5	476.27
		Difference	-68.83	-14.5	-415	72	-45	-6.5	142.31
		Difference significance	*Z* = 2.275, *p* = 0.023						
	INSECTS	I.V.	792.17	692	373	1716	504	1011	383.04
		M.V.	630.33	541.5	294	1664	442	698.5	363.36
		Difference	-120.25	-63	-473	129	-224.5	-4	173.86
		Difference significance	*Z* = 2.275, *p* = 0.023						
BF-10	CENTER	I.V.	413.58	417.5	280	568	347	463.5	83.57
		M.V.	427.92	418	276	695	317	486.5	131.69
		Difference	14.33	23	-116	237	-34	35.5	86.38
		Difference significance	*Z* = 0.549, *p* = 0.583						
	BALANCE	I.V.	446.08	445	220	674	335.5	549	138.46
		M.V.	448.00	407.5	212	845	328	528.5	176.75
		Difference	1.92	1.5	-197	229	-14.5	26.5	97.63
		Difference significance	*Z* = 0.275, *p* = 0.784						
	INSECTS	I.V.	472.83	439	309	780	383.5	549.5	133.60
		M.V.	364.17	364.5	196	534	293	430.5	94.53
		Difference	-64.42	-30	-259	54	-162.5	25	109.06
		Difference significance	*Z* = 1.451, *p* = 0.147						
Whole group:	CENTER	I.V.	464.58	380	79	1967	242.5	577	343.02
		M.V.	494.04	424.5	64	1828	288	629.5	332.94
		Difference	29.46	30	-649	251	-9.5	62	118.11
		Difference significance	*Z* = 3.517, *p* = 0.0004						
	BALANCE	I.V.	524.81	424	82	1849	226	674.5	421.57
		M.V.	509.43	398.5	63	1759	264	655.5	378.37
		Difference	-15.38	-5	-415	260	-24	31.5	108.63
		Difference significance	*Z* = 0.676, *p* = 0.498						
	INSECTS	I.V.	664.24	492.5	103	2698	346.5	820.5	512.26
		M.V.	543.86	413	87	2300	274	701.5	441.66
		Difference	-25.06	-9.5	-473	302	-104.5	54.5	144.10
		Difference significance	*Z* = 1.252, *p* = 0.211						

Legend: CENTER: relaxation training module; BALANCE: concentration training module; INSECTS: self-regulation training module; I.V.: initial measurement value (kOhm); M.V.: mean measurement value for the entire task (kOhm); Difference: a difference between I.V. and M.V.; *Z*: a result of the Wilcoxon signed-rank test; *p*: statistical significance. BF-2, BF-3, BF-7, BF-8, BF-9, BF-10: randomly selected patients from the Biofeedback group (patient code).

**Table 3:** Therapy effects and the type of change that occurred in the studied group by training module.

Patient: CODE	Change type	CENTER		BALANCE		INSECTS	
		Number of measurements	%	Number of measurements	%	Number of measurements	%
BF-2 (12 measurements)	Relaxation (increase in value)	7	58.33	3	25.00	1	8.33
	No change in value	0	0.00	0	0.00	1	8.33
	Activation (decrease in value)	5	41.67	9	75.00	10	83.33
BF-3 (12 measurements)	Relaxation (increase in value)	7	58.33	3	25.00	9	75.00
	Activation (decrease in value)	5	41.67	9	75.00	3	25.00
BF-7 (12 measurements)	Relaxation (increase in value)	11	91.67	12	100.00	10	83.33
	Activation (decrease in value)	1	8.33	0	0.00	2	16.67
BF-8 (12 measurements)	Relaxation (increase in value)	10	83.33	7	58.33	4	33.33
	Activation (decrease in value)	2	16.67	5	41.67	8	66.67
BF-9 (12 measurements)	Relaxation (increase in value)	8	66.67	1	8.33	3	25.00
	Activation (decrease in value)	4	33.33	11	91.67	9	75.00
BF-10 (12 measurements)	Relaxation (increase in value)	8	66.67	7	58.33	5	41.67
	Activation (decrease in value)	4	33.33	5	41.67	7	58.33
Total (72 measurements)	Relaxation (increase in value)	51	70.83	33	45.83	32	44.44
	No change in value	0	0.00	0	0.00	1	1.39
	Activation (decrease in value)	21	29.17	39	54.17	39	54.17

**Table 4:** Trend determined on the basis of the mean I.V. measurements on a given day.

Patient CODE	*R*_*S*_	*p*
BF-2	0.769	0.003
BF-3	0.797	0.002
BF-7	0.692	0.013
BF-8	0.364	0.245
BF-9	0.825	0.001
BF-10	0.483	0.112

*R*_*S*_: Spearman's correlation coefficient; *p*: statistical significance.

## Data Availability

All data is available with the authors on reasonable request.
